# Gene prediction in eukaryotes with a generalized hidden Markov model that uses hints from external sources

**DOI:** 10.1186/1471-2105-7-62

**Published:** 2006-02-09

**Authors:** Mario Stanke, Oliver Schöffmann, Burkhard Morgenstern, Stephan Waack

**Affiliations:** 1 lnstitut für Mikrobiologie und Genetik, Universität Göttingen, Göttingen, Germany; 2 lnstitut für Numerische und Angewandte Mathematik, Universität Göttingen, Göttingen, Germany

## Abstract

**Background:**

In order to improve gene prediction, extrinsic evidence on the gene structure can be collected from various sources of information such as genome-genome comparisons and EST and protein alignments. However, such evidence is often incomplete and usually uncertain. The extrinsic evidence is usually not sufficient to recover the complete gene structure of all genes completely and the available evidence is often unreliable. Therefore extrinsic evidence is most valuable when it is balanced with sequence-intrinsic evidence.

**Results:**

We present a fairly general method for integration of external information. Our method is based on the evaluation of hints to potentially protein-coding regions by means of a *Generalized Hidden Markov Model *(GHMM) that takes both intrinsic and extrinsic information into account. We used this method to extend the *ab initio *gene prediction program *AUGUSTUS *to a versatile tool that we call *AUGUSTUS+*. In this study, we focus on hints derived from matches to an EST or protein database, but our approach can be used to include arbitrary user-defined hints. Our method is only moderately effected by the length of a database match. Further, it exploits the information that can be derived from the *absence *of such matches. As a special case, AUGUSTUS+ can predict genes under user-defined constraints, e.g. if the positions of certain exons are known. With hints from EST and protein databases, our new approach was able to predict 89% of the exons in human chromosome 22 correctly.

**Conclusion:**

Sensitive probabilistic modeling of extrinsic evidence such as sequence database matches can increase gene prediction accuracy. When a match of a sequence interval to an EST or protein sequence is used it should be treated as compound information rather than as information about individual positions.

## Background

Finding protein-coding genes in eukaryotic genomic sequences with *in-silico *methods remains an important challenge in computational genomics, despite many years of intensive research work. Existing methods fall into two groups with respect to the data they utilize. The first group consists of *ab initio *programs which use only the query genomic sequence as input. Examples are the programs GENSCAN [1], AUGUSTUS [2] and HMMGene [3] which are HMM-based and GENEID [4]. The second group of gene-finding methods, extrinsic methods, comprises all programs which use data other than the query genomic sequence. Some extrinsic methods use genomic sequences from other species. A cross-species comparison of genomic sequences can help in predicting genes in the given query sequence. This approach is commonly referred to as *comparative gene prediction*. The corresponding programs need as additional input a collection of informant sequences (SGP2 [5], TWINSCAN [6]) or genomic sequences that are syntenic to the query sequence (N-SCAN [7], SLAM [8], DOUBLESCAN [9], AGenDA [10] or methods based on evolutionary Hidden Markov Models [11,12]). Other extrinsic methods use a database of known expressed sequences or proteins. Some of the best available *ab initio *gene prediction programs have been extended and have become more accurate by exploiting additional *extrinsic *data sources such as protein or EST sequences [13]. GENSCAN, for example, has an extension called GenomeScan that uses BLAST alignments with protein sequences [14]. HMMGene, has an extension that integrates into the HMM information from BLAST alignments of the query sequence with cDNA, EST and protein sequences [15]. Another program which uses extrinsic evidence is Gene Wise. It aligns a protein sequence to a genomic sequence [16].

Further, ExonHunter allows, like the method presented here, to integrate hints from both cross-species comparison and protein and EST databases [17]. Besides the two groups of programs described above, there are also programs like Combiner [18] that combine the predictions of several individual gene finders. Most of the above mentioned programs use statistical models of sub-structures of genes such as splice sites, exons or introns to derive evidences about the gene structure. All evidences, which may well contradict one another, must be combined to produce one output gene structure. A major challenge in computational gene prediction is therefore to design good statistical models that are able to deal with different types of information from heterogeneous sources.

In this paper, we introduce a gene finding approach that combines *intrinsic information *about a genomic sequence *s *with *extrinsic information*. In general, extrinsic information is evidence about the gene structure of the DNA sequence *s *that comes from a source other than the intrinsic model. Typically, such information can be gathered by comparing *s *to other sequences such as ESTs or a second syntenic genomic sequence from another species. We also consider expert knowledge to be extrinsic information, for example experimentally verified introns. Further, sequence analysis complementary to the intrinsic model may provide extrinsic information. For example, an external CpG island finder can find evidence that is not accounted for by the HMM and provide hints on a gene start. In this study, we present an application where EST and protein databases are used as source of extrinsic information.

In this study we present a stochastic model for gene prediction that generalizes the previously introduced GHMM AUGUSTUS [2] in a natural way by incorporating hints from external sources. Our method may be applied to hints from various sources; we also developed an application in the setting of comparative gene prediction. Hints from different sources can be used simultaneously, taking into account their different levels of reliability.

Our model considers hints that say that an interval of *s *is likely to be *part *of a possibly larger exon as well as hints that say that an interval is likely to be a *complete *exon. In addition to an increased sensitivity the specificity of the prediction is increased, because a search for hints that gave no results in a certain region may lead to a correction of a false positive exon with AUGUSTUS+. This is so because the absence of hints about coding regions slightly favors the prediction of non-coding regions.

### Combining intrinsic and extrinsic information for gene prediction

A GHMM for gene prediction is a probabilistic model of a random DNA sequence and its random gene structure. It assigns to each pair of a DNA-sequence *s *and a gene structure *φ *the *joint probability P*(*φ*, *s*). Based on this model, GHMM-based gene prediction programs find for a given input DNA sequence *s *the gene structure *φ *that is most likely given *s *– in other words, they find the gene structure *φ *that maximizes the *conditional probability P*(*φ *| *s*) which is called the *a-posteriori *probability of *φ*. Maximizing *P*(*φ *| *s*) for a fixed sequence *s *is equivalent to maximizing *P*(*φ*, *s*) which can be done using the well known Viterbi algorithm.

### A GHMM which emits a DNA sequence *and *hints to the gene structure

In this subsection, we explain how extrinsic information is integrated in the GHMM of AUGUSTUS. We call an individual piece of extrinsic information about the input DNA sequence a *hint*. For example, a potential splice site position derived from an alignment of *s *with an EST sequence is a hint. We distinguish 6 types of hints, namely hints to a translation start site, a stop codon, a donor (5') splice site or an acceptor (3') splice site, hints to a coding region and hints to an exon. Let

TYPE = {*start, stop, dss, ass, exonpart, exon*}

denote the set of hint types. Each hint refers to either the forward or to the reverse strand. For example a hint of type *start *would suggest that the coding region of a gene starts on a certain strand at a certain position in the input sequence. A hint of type *exonpart *suggests that a certain interval *α *of the input sequence is part of an exon, i.e. an exon either properly contains this interval *α *or is equal to it. In addition, an *exonpart *hint specifies a reading frame and a strand. Note that a hint that suggests that a single position is coding, as it is used in GenomeScan, is the special case of an *exonpart *hint of length 1. A hint of type *exon *specifies the strand, the reading frame and the *exact *start and end position of an exon. We assign to each hint a *grade g *which allows to distinguish different levels of confidence of the hints according to their respective source of information. AUGUSTUS+ uses currently four different grades to distinguish different sources of hints: manual (user-defined), EST alignment, protein alignment and combined hints, i.e. EST hints confirmed by a protein match.

### Extended model

**AUGUSTUS+. **We extend the GHMM used in AUGUSTUS to a GHMM that considers the above types of hints for gene prediction. Let *h *be the collection of all hints, which have been obtained for the input DNA sequence *s*. In the extended model not only the gene structure *φ*' and the DNA sequence *s *are thought to be the result of a random process but also the collection of hints *h*. This way, we obtain a probability distribution on the set of all triples (*φ*, *s*, *h*) of a gene structure *φ*, a DNA sequence *s *and a collection *h *of hints. AUGUSTUS+ assigns to each such triple a joint probability

*P*(*φ*, *s*, *h*)     (1)

and then finds the gene structure *φ *that maximizes the *conditional *probability (or *a-posteriori *probability) *P*(*φ *| *s*, *h*) for given *s *and *h*, which is again equivalent to maximizing the joint probability (1).

In order that the new model is still a GHMM, we combine the DNA sequence *s *over the alphabet Σ = {a, c, g, t} and the collection of hints *h *to one sequence *s' *over a suitably chosen larger alphabet Σ' which we define below. Accordingly, our extended model generates a random sequence *s' *which contains both a random DNA sequence *s *and a random set of hints *h*. The emissions of the new GHMM are from the extended alphabet Σ', and according to (1), we maximize *P*(*φ*, *s'*) = *P*(*φ*, *s*, *h*). As in the original model used in AUGUSTUS, a maximum a-posteriori gene structure and its a-posteriori probability can be calculated with the usual *Viterbi *and *forward *algorithm, respectively. The new model is again a GHMM which can be though of as a random generative process producing alternately states and strings. However, we will not prove that claim here and refer to [19].

Each hint about the gene structure of the DNA sequence *s *is associated with a certain position in *s*. For hints of type *start, stop, dss *and ass, this is natural as these hints contain information associated with a single position. Hints of type *exonpart *or *exon*, though, contain information about a *range *of positions in *s*, say from *a *to *b*. In this case we associate the hint with the right-end position *b*. The *i*-th symbol of the string *s' *specifies the nucleotide at position *i *of *s and *all hints which are associated with *i*. At most one hint of each type can be associated with each position.

Formally, we define a collection *h *of hints for a sequence *s *as a tuple

*h *= (*h*_*i*, *t *_: 1 ≤ *i *≤ |*s*|, *t *∈ TYPE)

where *h*_*i*, *t *_is the hint of type *t *at position *i*. If no hint of type *t *exists at position *i*, we write *h*_*i*, *t *_= ⋔, i.e. the pitchfork symbol stands for 'no hint'. The extended emission alphabet is a Cartesian product

Σ' = {a, c, g, t} × *H*_start _× *H*_stop _× … × *H*_exon_.

The *i*-th character of sequence *s' *is of the form (*b*_*i*_, *h*_*i*, *start*_, *h*_*i*, *stop*_, ..., *h*_*i*, *exon*_). Here, *b*_*i *_is the nucleotide at position *i *and *h*_*i*, *start *_to *h*_*i*, *exon *_specify the hints of type *start*, *stop*, ..., *exon*, respectively, associated with *i *if there are any. We exemplify this with the *start *hints and the *exonpart *hints. *h*_*i*, *start *_specifies a *start *hint at position *i*. If there is no hint about a translation start at position *i *then *h*_*i*, *start *_= ⋔. This is in practice the most common case. Otherwise, *h*_*i*, *start *_= (grade, strand), where 'grade' is the grade of the hint and 'strand' is either the forward or reverse strand, depending on the reading direction of the putative gene. *h*_*i*, *stop*_, *h*_*i*, *dss *_and *h*_*i*, *ass *_are analogously defined by the *stop*, *dss *and *ass *hints for position *i*. Consider as second example the *exonpart *hint at position *i*. If there is no *exonpart *hint ending at position *i *then *h*_*i*, *exonpart *_= ⋔. Otherwise *h*_*i*, *exonpart*_*= *(grade, strand, length, reading frame), 'length' is the length of the *exonpart *hint range. In other words, while the *exonpart *hint is associated with its right-end position, its length and therefore implicitly the left-end position is part of the information encoded in the emitted character. 'reading frame' is one of the three possible reading frames. The *exon *hints are defined analogously to the *exonpart *hints.

Another way of thinking about this is that the GHMM produces output on 7 tapes, the first tape contains the DNA bases, the second tape contains the hints about start sites and the letters on this tape are from an alphabet *H*_start_, similarly the third to seventh tape contain the hints of the remaining five types, each having its own alphabet. Note that the letters on all but the first tape are themselves tuples. This choice of the extended emission alphabet Σ' and its interpretation ensures that all hints can be summarized together with the input DNA sequence in one sequence *s'*, as is required for an emission of a GHMM. This definition follows the principle: Every piece of information that is used for the prediction must also be emitted by the GHMM.

### The probabilities of hints

In order to describe the model AUGUSTUS+ we need to specify the probability distribution (1). The probabilities of gene structures *φ *and DNA-sequences *s *is the same as in AUGUSTUS [19]; the same transition probabilities are used and exactly the same models for the DNA sequence. In the present paper, we make the general simplifying assumption that hints emitted at different positions *i *of a sequence *s *are *independent *given the gene structure *φ *and the DNA-sequence *s*. Further, the hints of different types *t *emitted at the same position *i *are assumed to be independent. Thus, we have

P(φ,s,h)=P(φ,s)⋅P(h|φ,s)=P(φ,s)⋅∏1≤i≤|s|t∈TYPEP(hi,t|φ,s).     (2)
 MathType@MTEF@5@5@+=feaafiart1ev1aaatCvAUfKttLearuWrP9MDH5MBPbIqV92AaeXatLxBI9gBaebbnrfifHhDYfgasaacH8akY=wiFfYdH8Gipec8Eeeu0xXdbba9frFj0=OqFfea0dXdd9vqai=hGuQ8kuc9pgc9s8qqaq=dirpe0xb9q8qiLsFr0=vr0=vr0dc8meaabaqaciaacaGaaeqabaqabeGadaaakeaafaqadeGabaaabaGaemiuaaLaeiikaGccciGae8NXdyMaeiilaWIaem4CamNaeiilaWIaemiAaGMaeiykaKIaeyypa0JaemiuaaLaeiikaGIae8NXdyMaeiilaWIaem4CamNaeiykaKIaeyyXICTaemiuaaLaeiikaGIaemiAaGMaeiiFaWNae8NXdyMaeiilaWIaem4CamNaeiykaKcabaGaeyypa0JaemiuaaLaeiikaGIae8NXdyMaeiilaWIaem4CamNaeiykaKIaeyyXIC9aaebuaeaacqWGqbaucqGGOaakcqWGObaAdaWgaaWcbaGaemyAaKMaeiilaWIaemiDaqhabeaakiabcYha8jab=z8aMjabcYcaSiabdohaZjabcMcaPaWcbaqbaeqabiqaaaqaaiabigdaXiabgsMiJkabdMgaPjabgsMiJkabcYha8jabdohaZjabcYha8bqaaiabdsha0jabgIGiolabbsfaujabbMfazjabbcfaqjabbweafbaaaeqaniabg+GivdaaaOGaeiOla4IaaCzcaiaaxMaadaqadaqaaiabikdaYaGaayjkaiaawMcaaaaa@7A0F@

In order to define the conditional probability *P*(*h*_*i*, *t *_| *φ*, *s*) – i.e. the probability of the observed hint of type *t *at position *i *given the gene structure *φ *and the sequence *s *– we introduce two terms: We say that a hint is *compatible with a gene structure φ *if *φ *obeys the extrinsic information given by the hint. For example, a *start *hint at a certain position is compatible with any gene structure in which a coding region starts at this position on the strand specified by the hint. In case the true gene structure is known a hint that is compatible with it is said to be *correct*. Next, we say that a hint *h*_*i*, *t *_is *compatible with the sequence s *if a minimal biological consistency requirement is satisfied. The meaning of this requirement depends on the type of the hint. A *start *hint is compatible with the sequence if and only if an ATG ends at the position *i *and the hint is on the forward strand or the reverse complement of ATG ends at that position and the hint is on the reverse strand. Analogously a *stop *hint requires the presence of a stop codon in the sequence and a *dss *and *ass *hint require the presence of the dinucleotide consensus of 'GU-AG' introns. An *exonpart *hint is compatible with *s *if and only if the interval contains no stop codon in the corresponding reading frame on the corresponding strand. Finally, a hint of type *exon *is only compatible with *s *if the interval contains no in-frame stop codon on the specified strand *and *the interval is bounded by splice and/or start/stop codons in a biologically meaningful way.

In the AUGUSTUS model every gene structure that violates above minimal biological consistency requirement is impossible and has probability 0. Therefore, every hint that is compatible with some possible gene structure on *s *is also compatible with the sequence *s*. AUGUSTUS+ considers only hints which are compatible with the input sequence *s*; non-compatible hints are ignored. Thus there are three cases for a hint, given the sequence *s *and a possible gene structure *φ*. Firstly, it can be compatible with the gene structure *φ *and therefore compatible with *s*. Secondly, it can be compatible with *s *but not with the gene structure *φ*, and thirdly, it can be incompatible with both *φ *and *s*.

We make the general simplifying assumption that for a hint *h*_*i*, *t *_≠ ⋔ the probability *P*(*h*_*i*, *t *_| *φ*, *s*) *depends only on the type t of the hint, its grade g, whether it is compatible with φ and whether it is compatible with s*. Thus, for all grades *g *and types *t *there are numerical values *q*^+^(*t*, *g*), *q*^-^(*t*, *g*) such that

P(hi,t|φ,s)={q+(t,g)if hi,t is compatible with φ;q−(t,g)if hi,t is compat. with s but not with φ;0if hi,t is not compatible with s.     (3)
 MathType@MTEF@5@5@+=feaafiart1ev1aaatCvAUfKttLearuWrP9MDH5MBPbIqV92AaeXatLxBI9gBaebbnrfifHhDYfgasaacH8akY=wiFfYdH8Gipec8Eeeu0xXdbba9frFj0=OqFfea0dXdd9vqai=hGuQ8kuc9pgc9s8qqaq=dirpe0xb9q8qiLsFr0=vr0=vr0dc8meaabaqaciaacaGaaeqabaqabeGadaaakeaacqWGqbaucqGGOaakcqWGObaAdaWgaaWcbaGaemyAaKMaeiilaWIaemiDaqhabeaakiabcYha8HGaciab=z8aMjabcYcaSiabdohaZjabcMcaPiabg2da9maaceaabaqbaeaabmGaaaqaaiabdghaXnaaCaaaleqabaGaey4kaScaaOGaeiikaGIaemiDaqNaeiilaWIaem4zaCMaeiykaKcabaGaeeyAaKMaeeOzayMaeeiiaaIaemiAaG2aaSbaaSqaaiabdMgaPjabcYcaSiabdsha0bqabaGccqqGGaaicqqGPbqAcqqGZbWCcqqGGaaicqqGJbWycqqGVbWBcqqGTbqBcqqGWbaCcqqGHbqycqqG0baDcqqGPbqAcqqGIbGycqqGSbaBcqqGLbqzcqqGGaaicqqG3bWDcqqGPbqAcqqG0baDcqqGObaAcqqGGaaicqWFgpGzcqGG7aWoaeaacqWGXbqCdaahaaWcbeqaaiabgkHiTaaakiabcIcaOiabdsha0jabcYcaSiabdEgaNjabcMcaPaqaaiabbMgaPjabbAgaMjabbccaGiabdIgaOnaaBaaaleaacqWGPbqAcqGGSaalcqWG0baDaeqaaOGaeeiiaaIaeeyAaKMaee4CamNaeeiiaaIaee4yamMaee4Ba8MaeeyBa0MaeeiCaaNaeeyyaeMaeeiDaqNaeeOla4IaeeiiaaIaee4DaCNaeeyAaKMaeeiDaqNaeeiAaGMaeeiiaaccbiGae43CamNaeeiiaaIaeeOyaiMaeeyDauNaeeiDaqNaeeiiaaIaeeOBa4Maee4Ba8MaeeiDaqNaeeiiaaIaee4DaCNaeeyAaKMaeeiDaqNaeeiAaGMaeeiiaaIae8NXdyMaei4oaSdabaGaeGimaadabaGaeeyAaKMaeeOzayMaeeiiaaIaemiAaG2aaSbaaSqaaiabdMgaPjabcYcaSiabdsha0bqabaGccqqGGaaicqqGPbqAcqqGZbWCcqqGGaaicqqGUbGBcqqGVbWBcqqG0baDcqqGGaaicqqGJbWycqqGVbWBcqqGTbqBcqqGWbaCcqqGHbqycqqG0baDcqqGPbqAcqqGIbGycqqGSbaBcqqGLbqzcqqGGaaicqqG3bWDcqqGPbqAcqqG0baDcqqGObaAcqqGGaaicqGFZbWCcqqGUaGlaaaacaGL7baacaWLjaGaaCzcamaabmaabaGaeG4mamdacaGLOaGaayzkaaaaaa@D0BC@

The probabilities *q*^+^(*t*, *g*), *q*^-^(*t*, *g*) have been estimated on a part of our training set with known gene structures and given hints. Example for the estimation: For 386 of the 500 translation starts in the training set we received a hint coming from a protein database search. Therefore we estimated

*q*^+^(*start*, *Protein*) = 386/500 ≈ 0.77.     (4)

We also had *Protein start *hints for 47 of the approximate 145000 ATGs which are not a translation start. Therefore, we estimated

*q*^-^(*start*, *Protein*) = 47/145000 ≈ 3.2·10^-4^.     (5)

This training directly assesses the reliability of the hints, not indirectly through the incorporation of BLAST output numbers with ad-hoc parameters. For details on the training of hints parameters see [19]. In the case of user defined constraints (*g *= *manual*) we simply set *q*^-^(*t*, *g*) = 0 for all types *t*, i.e. *manual *hints that are incompatible with the gene structure are never observed. This has the effect that *P*(*φ*, *s*, *h*) = 0 for every gene structure *φ *which is incompatible with a *manual *hint. Therefore AUGUSTUS+ predicts a gene structure which is compatible with all *manual *hints if that is possible with the model. These user constraints can also be set at the AUGUSTUS web server [20].

The simplifying independence assumption (2) is in some cases clearly violated. This is for example the case when the same EST alignment was used to generate a hint for an exon and also for the bordering splice sites. In this case we keep only those nonredundant hints which best summarize the extrinsic information: We keep the *exon *hint and delete the *dss *and *ass *hint. Also, the sets of *EST *and *Combined *hints are not stochastically independent, as those hints may in both cases depend on the same EST. In the case of identical hints of different types we keep only the one with the most reliable grade. These changes to the set of hints are made before the hints are considered for gene prediction.

### Reward for gene structures supported by hints

Suppose we have some (possibly empty) collection *h *of hints for an input DNA sequence *s*. We want to examine the effect that an additional hint not present in *h *has on the predicted gene structure. Clearly, gene structures that are compatible with the new hint should get some sort of bonus over ones that are incompatible with it. Let *h' *denote the collection of hints obtained by adding the new hint to *h*. Let the new hint be at position *i *of type *t *and with grade *g*, i.e. *h'*_*i'*, *t' *_= *h*_*i'*, *t' *_for all (*i'*, *t'*) ≠ (*i*, *t*) and *h'*_*i*, *t *_≠ *h*_*i*, *t *_= ⋔.

Suppose that *φ*^+ ^and *φ*^- ^are gene structures on *s *such that the new hint *h'*_*i*, *t *_is compatible with *φ*^+ ^but not with *φ*^- ^We obtain

P(φ+,s,h′)P(φ−,s,h′)=P(φ+,s,h)⋅q+(t,g)P(hi,t=⋔|φ+,s)P(φ−,s,h)⋅q−(t,g)P(hi,k=⋔|φ−,s)     (6)
 MathType@MTEF@5@5@+=feaafiart1ev1aaatCvAUfKttLearuWrP9MDH5MBPbIqV92AaeXatLxBI9gBaebbnrfifHhDYfgasaacH8akY=wiFfYdH8Gipec8Eeeu0xXdbba9frFj0=OqFfea0dXdd9vqai=hGuQ8kuc9pgc9s8qqaq=dirpe0xb9q8qiLsFr0=vr0=vr0dc8meaabaqaciaacaGaaeqabaqabeGadaaakeaadaWcaaqaaiabbcfaqjabcIcaOGGaciab=z8aMnaaCaaaleqabaGaey4kaScaaOGaeiilaWIaem4CamNaeiilaWIafmiAaGMbauaacqGGPaqkaeaacqqGqbaucqGGOaakcqWFgpGzdaahaaWcbeqaaiabgkHiTaaakiabcYcaSiabdohaZjabcYcaSiqbdIgaOzaafaGaeiykaKcaaiabg2da9maalaaabaGaeeiuaaLaeiikaGIae8NXdy2aaWbaaSqabeaacqGHRaWkaaGccqGGSaalcqWGZbWCcqGGSaalcqWGObaAcqGGPaqkcqGHflY1daWcaaqaaiabdghaXnaaCaaaleqabaGaey4kaScaaOGaeiikaGIaemiDaqNaeiilaWIaem4zaCMaeiykaKcabaGaemiuaaLaeiikaGIaemiAaG2aaSbaaSqaaiabdMgaPjabcYcaSiabdsha0bqabaGccqGH9aqptuuDJXwAK1uy0HMmaeHbfv3ySLgzG0uy0HgiuD3BaGabaiab+rTiJlabcYha8jab=z8aMnaaCaaaleqabaGaey4kaScaaOGaeiilaWIaem4CamNaeiykaKcaaaqaaiabbcfaqjabcIcaOiab=z8aMnaaCaaaleqabaGaeyOeI0caaOGaeiilaWIaem4CamNaeiilaWIaemiAaGMaeiykaKIaeyyXIC9aaSaaaeaacqWGXbqCdaahaaWcbeqaaiabgkHiTaaakiabcIcaOiabdsha0jabcYcaSiabdEgaNjabcMcaPaqaaiabdcfaqjabcIcaOiabdIgaOnaaBaaaleaacqWGPbqAcqGGSaalcqWGRbWAaeqaaOGaeyypa0Jae4h1ImUaeiiFaWNae8NXdy2aaWbaaSqabeaacqGHsislaaGccqGGSaalcqWGZbWCcqGGPaqkaaaaaiaaxMaacaWLjaWaaeWaaeaacqaI2aGnaiaawIcacaGLPaaaaaa@9CCB@

This equation holds because using (2) the probabilities P(*φ*^+^, *s*, *h'*) and P(*φ*^+^, *s*, *h*) differ only by the two factors *q*^+^(*t*, *g*) and *P*(*h*_*i*, *t *_= ⋔ | *φ*^+^, *s*) as the new hint is compatible with *φ*^+^. Likewise, the denominators on both sides of this equation are equal. The interpretation of (6) is that introducing a hint of grade *g *and type *t *gives the a-posteriori probabilities of all gene structures which are compatible with that hint a relative bonus of

q+(t,g)q−(t,g)⋅P(hi,t=⋔|φ−,s)P(hi,t=⋔|φ+,s)     (7)
 MathType@MTEF@5@5@+=feaafiart1ev1aaatCvAUfKttLearuWrP9MDH5MBPbIqV92AaeXatLxBI9gBaebbnrfifHhDYfgasaacH8akY=wiFfYdH8Gipec8Eeeu0xXdbba9frFj0=OqFfea0dXdd9vqai=hGuQ8kuc9pgc9s8qqaq=dirpe0xb9q8qiLsFr0=vr0=vr0dc8meaabaqaciaacaGaaeqabaqabeGadaaakeaadaWcaaqaaiabdghaXnaaCaaaleqabaGaey4kaScaaOGaeiikaGIaemiDaqNaeiilaWIaem4zaCMaeiykaKcabaGaemyCae3aaWbaaSqabeaacqGHsislaaGccqGGOaakcqWG0baDcqGGSaalcqWGNbWzcqGGPaqkaaGaeyyXIC9aaSaaaeaacqWGqbaucqGGOaakcqWGObaAdaWgaaWcbaGaemyAaKMaeiilaWIaemiDaqhabeaakiabg2da9mrr1ngBPrwtHrhAYaqeguuDJXwAKbstHrhAGq1DVbaceaGae8h1ImUaeiiFaWhcciGae4NXdy2aaWbaaSqabeaacqGHsislaaGccqGGSaalcqWGZbWCcqGGPaqkaeaacqWGqbaucqGGOaakcqWGObaAdaWgaaWcbaGaemyAaKMaeiilaWIaemiDaqhabeaakiabg2da9iab=rTiJlabcYha8jab+z8aMnaaCaaaleqabaGaey4kaScaaOGaeiilaWIaem4CamNaeiykaKcaaiaaxMaacaWLjaWaaeWaaeaacqaI3aWnaiaawIcacaGLPaaaaaa@7089@

over those which do not respect that hint. For example, this means that when the protein search lead to a hint about a putative translation start site, then gene structures which have a translation start site at this position become more likely. Using (4) and (5) the probability of a gene structure *with *that start site relative to the probability of a gene structure *without *that start site increases by approximately a factor of 0.773.2⋅10−4⋅10.23≈10462
 MathType@MTEF@5@5@+=feaafiart1ev1aaatCvAUfKttLearuWrP9MDH5MBPbIqV92AaeXatLxBI9gBaebbnrfifHhDYfgasaacH8akY=wiFfYdH8Gipec8Eeeu0xXdbba9frFj0=OqFfea0dXdd9vqai=hGuQ8kuc9pgc9s8qqaq=dirpe0xb9q8qiLsFr0=vr0=vr0dc8meaabaqaciaacaGaaeqabaqabeGadaaakeaadaWcaaqaaiabicdaWiabc6caUiabiEda3iabiEda3aqaaiabiodaZiabc6caUiabikdaYiabgwSixlabigdaXiabicdaWmaaCaaaleqabaGaeyOeI0IaeGinaqdaaaaakiabgwSixpaalaaabaGaeGymaedabaGaeGimaaJaeiOla4IaeGOmaiJaeG4mamdaaiabgIKi7kabigdaXiabicdaWiabisda0iabiAda2iabikdaYaaa@4705@. An additional hint may turn a compatible gene structure into the maximum a-posteriori gene structure.

### Penalty for gene parts not supported by hints

A 'bonus effect' as above was clearly intended when we constructed the new model. It turns out that the extended probabilistic model entails another effect, which could be called a 'malus effect'. Consider the following example. Suppose that a search for hints has produced no results (*h*_*i*, *t *_= ⋔ for all *i*, *t*) on a certain piece of DNA *s*. Imagine that *s *is a small part of a longer input DNA sequence. Let *φ*_0 _and *φ*_1 _be any two gene structures. Then

P(φ1|s,h)P(φ0|s,h)=P(φ1,s,h)P(φ0,s,h)=P(φ1,s)⋅P(h|φ1,s)P(φ0,s)⋅P(h|φ0,s)=P(φ1|s)P(φ0|s)⋅∏i,tP(hi,t=⋔|φ1,s)P(hi,t=⋔|φ0,s)     (8)
 MathType@MTEF@5@5@+=feaafiart1ev1aaatCvAUfKttLearuWrP9MDH5MBPbIqV92AaeXatLxBI9gBaebbnrfifHhDYfgasaacH8akY=wiFfYdH8Gipec8Eeeu0xXdbba9frFj0=OqFfea0dXdd9vqai=hGuQ8kuc9pgc9s8qqaq=dirpe0xb9q8qiLsFr0=vr0=vr0dc8meaabaqaciaacaGaaeqabaqabeGadaaakeaafaqadeGabaaabaWaaSaaaeaacqqGqbaucqGGOaakiiGacqWFgpGzdaWgaaWcbaGaeGymaedabeaakiabcYha8jabdohaZjabcYcaSiabdIgaOjabcMcaPaqaaiabbcfaqjabcIcaOiab=z8aMnaaBaaaleaacqaIWaamaeqaaOGaeiiFaWNaem4CamNaeiilaWIaemiAaGMaeiykaKcaaiabg2da9maalaaabaGaeeiuaaLaeiikaGIae8NXdy2aaSbaaSqaaiabigdaXaqabaGccqGGSaalcqWGZbWCcqGGSaalcqWGObaAcqGGPaqkaeaacqqGqbaucqGGOaakcqWFgpGzdaWgaaWcbaGaeGimaadabeaakiabcYcaSiabdohaZjabcYcaSiabdIgaOjabcMcaPaaacqGH9aqpdaWcaaqaaiabbcfaqjabcIcaOiab=z8aMnaaBaaaleaacqaIXaqmaeqaaOGaeiilaWIaem4CamNaeiykaKIaeyyXIC9exLMBbXgBcf2CPn2qVrwzqf2zLnharyGvLjhzH5wyaGabciaa+bfacqGGOaakcqWGObaAcqGG8baFcqWFgpGzdaWgaaWcbaGaeGymaedabeaakiabcYcaSiabdohaZjabcMcaPaqaaiabbcfaqjabcIcaOiab=z8aMnaaBaaaleaacqaIWaamaeqaaOGaeiilaWIaem4CamNaeiykaKIaeyyXICTaa4huaiabcIcaOiabdIgaOjabcYha8jab=z8aMnaaBaaaleaacqaIWaamaeqaaOGaeiilaWIaem4CamNaeiykaKcaaaqaaiabg2da9maalaaabaGaeeiuaaLaeiikaGIae8NXdy2aaSbaaSqaaiabigdaXaqabaGccqGG8baFcqWGZbWCcqGGPaqkaeaacqqGqbaucqGGOaakcqWFgpGzdaWgaaWcbaGaeGimaadabeaakiabcYha8jabdohaZjabcMcaPaaacqGHflY1daqeqbqaamaalaaabaGaemiuaaLaeiikaGIaemiAaG2aaSbaaSqaaiabdMgaPjabcYcaSiabdsha0bqabaGccqGH9aqptuuDJXwAK1uy0HMmaeXbfv3ySLgzG0uy0HgiuD3BaGqbaiab9rTiJlabcYha8jab=z8aMnaaBaaaleaacqaIXaqmaeqaaOGaeiilaWIaem4CamNaeiykaKcabaGaemiuaaLaeiikaGIaemiAaG2aaSbaaSqaaiabdMgaPjabcYcaSiabdsha0bqabaGccqGH9aqpcqqFulY4cqGG8baFcqWFgpGzdaWgaaWcbaGaeGimaadabeaakiabcYcaSiabdohaZjabcMcaPaaaaSqaaiabdMgaPjabcYcaSiabdsha0bqab0Gaey4dIunaaaGccaWLjaGaaCzcamaabmaabaGaeGioaGdacaGLOaGaayzkaaaaaa@D97D@

Now, let for example *φ*_0 _be a gene structure which has no exons in *s *and let *φ*_1 _be a gene structure with exactly one internal exon in *s*. Then in the product in (8) all probabilities of observing no hint given the one or the other gene structure cancel out except for the splice site hint types at the two positions where *φ*i has a splice site, for the *exon *type at the right end point of the exon and for the *exonpart *type at all ℓ coding positions. For these pairs of *i *and *t *the probability of observing a hint of type *t *at position *i *is larger given *φ*_1 _than given *φ*_0_, and thus the probability of observing no hint is smaller, i.e. *P*(*h*_*i*, *t *_= ⋔ | *φ*_1_, *s*) <*P*(*h*_*i*, *t *_= ⋔ | *φ*_0_, *s*). To give a concrete example, if the exon of *φ*_1 _has length 100, and the search for all hints has found no results, we would have

P(φ1|s,h)P(φ0|s,h)≈0.036⋅P(φ1|s)P(φ0|s).     (9)
 MathType@MTEF@5@5@+=feaafiart1ev1aaatCvAUfKttLearuWrP9MDH5MBPbIqV92AaeXatLxBI9gBaebbnrfifHhDYfgasaacH8akY=wiFfYdH8Gipec8Eeeu0xXdbba9frFj0=OqFfea0dXdd9vqai=hGuQ8kuc9pgc9s8qqaq=dirpe0xb9q8qiLsFr0=vr0=vr0dc8meaabaqaciaacaGaaeqabaqabeGadaaakeaadaWcaaqaaiabbcfaqjabcIcaOGGaciab=z8aMnaaBaaaleaacqaIXaqmaeqaaOGaeiiFaWNaem4CamNaeiilaWIaemiAaGMaeiykaKcabaGaeeiuaaLaeiikaGIae8NXdy2aaSbaaSqaaiabicdaWaqabaGccqGG8baFcqWGZbWCcqGGSaalcqWGObaAcqGGPaqkaaGaeyisISRaeGimaaJaeiOla4IaeGimaaJaeG4mamJaeGOnayJaeyyXIC9aaSaaaeaacqqGqbaucqGGOaakcqWFgpGzdaWgaaWcbaGaeGymaedabeaakiabcYha8jabdohaZjabcMcaPaqaaiabbcfaqjabcIcaOiab=z8aMnaaBaaaleaacqaIWaamaeqaaOGaeiiFaWNaem4CamNaeiykaKcaaiabc6caUiaaxMaacaWLjaWaaeWaaeaacqaI5aqoaiaawIcacaGLPaaaaaa@6128@

The quotient at the right hand side is the ratio of the a-posteriori probabilities of the two gene structures in the original *ab initio *AUGUSTUS model. Equation (9) means that in this case when AUGUSTUS+ is compared to AUGUSTUS the a-posteriori probability of a gene structure with an additional unsupported internal exon gets a relative malus factor of about 0.036 over gene structures which do not have the unsupported internal exon. Unsupported exons and splice sites are penalized, which seems reasonable: Experience shows that the largest part (more than three fourth) of the actual donor splice sites are supported by *EST dss *hints. Suppose a situation where the *ab initio *gene prediction program is 'in doubt' about this splice site in the sense that there are two likely gene structures with about equal a-posteriori probabilities. One gene structure has this splice site and one has not. In this situation an EST search that produced no hint makes the no-splice-site gene structure more likely. No information is also information. **Lengths of ***exonpart ***and ***exon ***hints. **We examined the information the length of a hint contains on its reliability and how it should be taken into consideration by a program. HMMGene can include, among others, BLAST hits to an EST database [15]. An EST hit of length ℓ indirectly has the following effect for HMMGene. The joint probability of the sequence and a parse in which a coding region covers the EST hit gets a relative bonus over the joint probability that of the sequence and a parse in which an intron or intergenic region covers the EST hit. This relative bonus is exponential in ℓ. With the ad-hoc parameters used in [15] it is approximately (1.2/1.07)^ℓ ^= 1.12^ℓ^. So, for example the relative bonus is about 300 when the EST hit has length 50 but it is as large as 7 10^9 ^when the EST hit has length 200. In the TWINSCAN model the length of a BLAST match has a similar effect. The relative bonus of a gene structure which has an exon covering a BLAST match of length ℓ over a gene structure which has an intron or intergenic region covering that match is also approximately exponential in ℓ. In the case of HMMGene with an EST search this length dependency has been identified as a problem by Krogh: "... ignoring a long EST is very improbable, whereas it is quite probable to ignore a short one.... for ESTs experimentation with other types of length dependences is necessary." In HMMGene "the specificity drops more than the sensitivity increases when ESTs are used." [15] For the case of *Protein *and *EST hints *obtained with AGRIPPA, we found that long *exonpart *hints are not much more often correct than shorter ones (data not shown, see [19]). In our model the relative bonus a gene structure gets for being compatible with a hint of type *exonpart *or *exon *does not depend on the length of the interval specified in the hint.

### Relevance of the p-value of a match

Another difference between our method and GenomeScan and HMMGene is that we do not make use of the BLAST p-value. A smaller p-value indicates that the BLAST hit of a given score is less likely to have occurred if the database consisted of random sequences not related to the query sequence. This fact seems to suggest to make use of the BLAST p-value and trust those hints more that are derived from a hit with a small p-value. Indeed, GenomeScan uses a heuristic in which the relative bonus of a gene structure is inversely proportional to the 10th root of the p-value. However, we found that the distribution of the e-value of correct *Protein exonpart *hints is about the same as the distribution of the e-value of incorrect *Protein exonpart *hints (data not shown, see [19]). Only very large e-values, say larger than 10^-10^, are somewhat more frequent among the incorrect hints than among the correct hints. This can be explained by the following presumption. Most of the incorrect *Protein exonpart *hints are not incorrect because the similarity between the genomic sequence and the protein sequence is a mere coincidence but because there are (strong) similarities to protein sequences in the nr database in regions of the genomic sequence which are not annotated as exons. The e-value and p-value contain only little information on the correctness of an exonpart *Protein *hint. Trying to exploit that information with AUGUSTUS+ by introducing different grades for higher e-values and lower e-values yielded no better results.

### Collecting Hints from EST and protein databases

The hints we here use as input to AUGUSTUS+ (in GFF format) are automatically generated by a program called AGRIPPA (The eponymous Roman general Agrippa was an adviser and close associate of the Roman emperor Augustus). AGRIPPA extends the local alignment search tool WU-BLAST [21]. It uses a protein sequence database and an EST database to infer hints about the coding regions in the input DNA sequence. Before such a database search is initiated, putative repetitive elements in the input DNA sequence are masked using the program RepeatMasker. When run on the protein database, AGRIPPA uses the resulting alignmens of a BLASTX search to infer *Protein *hints of all six types. When run on the EST database, it uses the resulting alignments of a BLASTN search (options: -Q 15 -R 15) to infer *EST *hints of the types *exonpart, exon, ass *and *dss*.

It should be mentioned that we make a systematic error with EST hints. It is due to the fact that ESTs can theoretically only be used to infer the mRNA sequence of a gene. Our method also finds presumable *non-coding *exons. The information which part of that mRNA sequence is coding cannot be derived from ESTs hits alone. Therefore, AGRIPPA has a program option, *Combined*, which tries to verify which parts of the partially reconstructed mRNA is coding by performing a protein database search with this sequence. After the EST database has been used to partially reconstruct the mRNA, each presumable part *τ *of an mRNA sequence is searched against the protein database. The parts of *τ *that are aligned to an amino acid sequence are relatively likely to be coding. Then the information from the original alignment of the ESTs to the input DNA sequence can be used to infer a partial presumable intron/exon structure. Again, a protein hit can be used to infer a translation start or stop site if the first or last amino acid of a protein has been aligned, respectively. Figure 2 shows an example for the construction of *Combined *hints.

**Figure 1:**
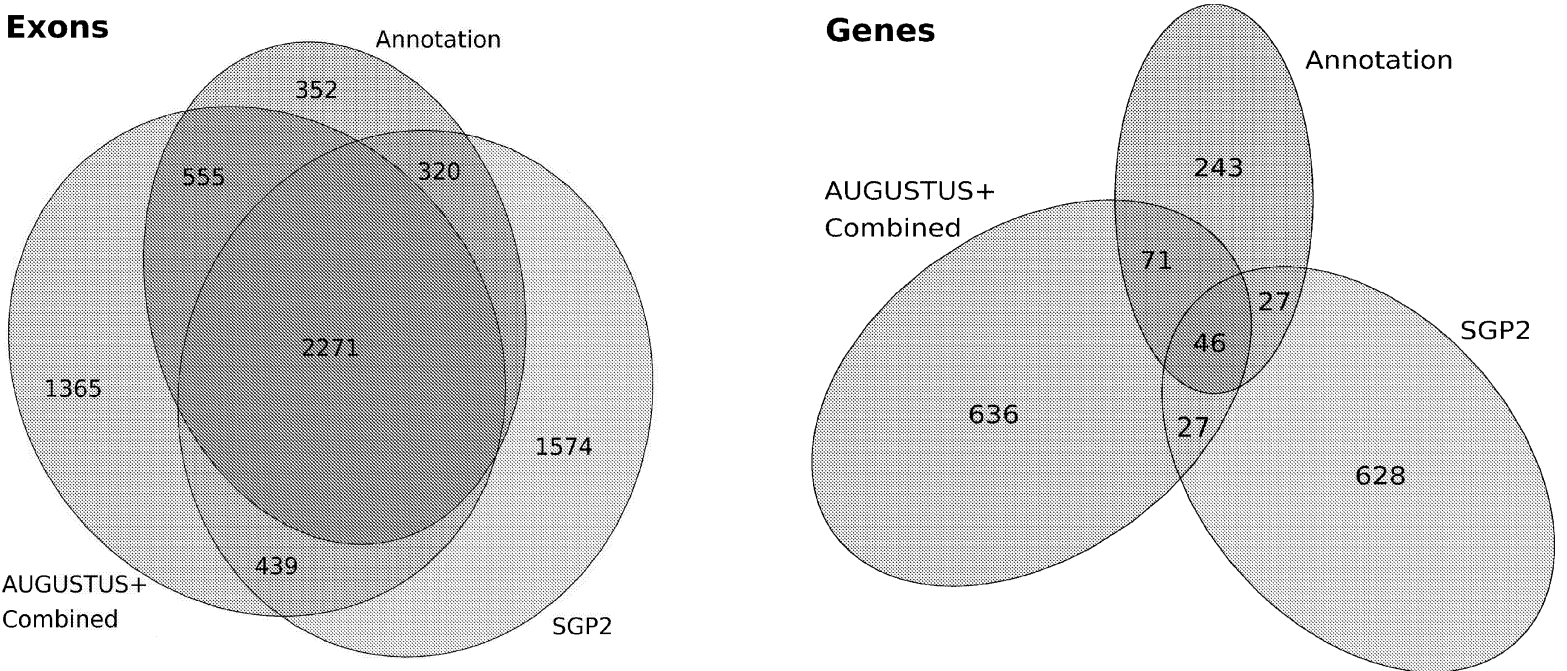
**Venn diagram of exons and genes**. Area-proportional Venn diagram of three sets of exons (top) and three sets of genes (bottom) for chromosome 22. 'Annotation' refers to the set of 387 genes compiled by the Sanger Institute. Examples: 2271 exons were in the Sanger Center annotation and were exactly predicted by AUGUSTUS+ using the *Combined *hints and by SGP2. The annotation set and the set of predictions of AUGUSTUS+ shared 71 genes identically, that were not in the set of SGP2 predictions.

**Figure 2:**
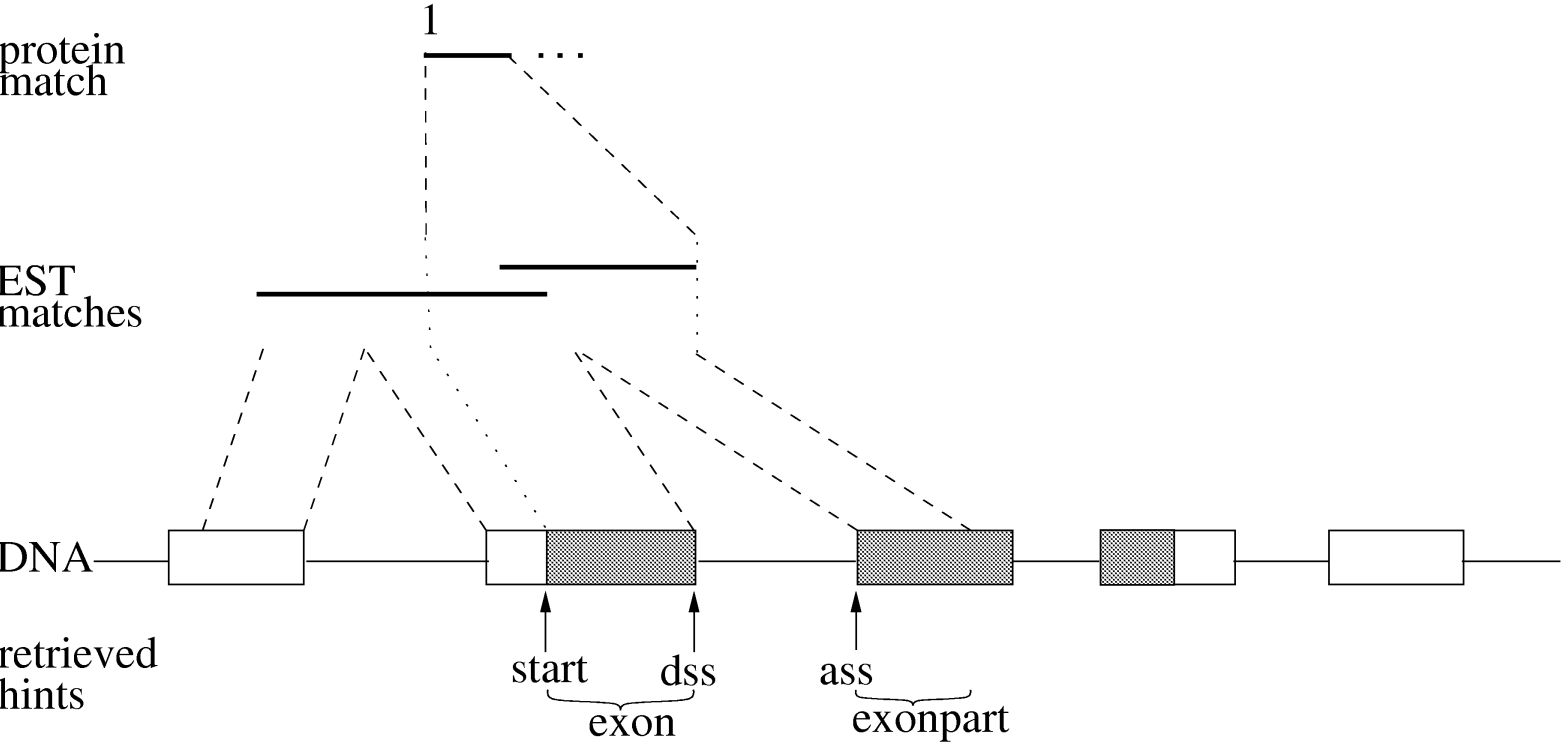
**Combined hints**. The information retrieved from a combination of EST and protein database searches. The input DNA sequence contains one gene of which the dark boxes are the coding parts. At first, ESTs matching the DNA sequence are found and clustered. The concatenation of the segments of the input DNA sequence which are aligned to the clustered ESTs is then searched against a protein database. The protein match can be used to infer which part of the EST consensus sequence was coding. In this example the alignment of the protein started at the first position of its amino acid sequence. Thus a likely translation start site (*start *hint) can be inferred.

## Test results

We tested AUGUSTUS+ on two different human test sets, the benchmark set *sag*l78 [22] and the well-annotated human chromosome 22 [23] and compared our results to a number of established gene finding programs which use different information sources for the prediction.

Guigó et. al. compiled the test set sag178 [22] which contains real genes and randomly generated intergenic sequence. This has the advantage that gene predictions in this area are certainly false positives, so the *specificity *of gene finding programs can better be assessed. Further, we tested AUGUSTUS+ with hints generated by our tool AGRIPPA in four different ways, (*a*) using the dbEST database, (*b*) using the non-redundant protein sequence database *nr *from NCBI, (*c*) using above described combination of an EST and protein search and (*d*) with all above hints together. We refer to these sources of hints as *Protein, EST, Combined *or *all hints*, respectively. In addition to some *ab initio *methods, we tested *GenomeScan *[14] which uses BLASTX alignments with protein sequences for the prediction and *TWINSCAN *[6], a reimplementation of GENSCAN that uses in this setting BLASTN alignments with the mouse genomic sequence. Furthermore, we compared our method to *SGP2 *[5], an extension of GENEID which here used TBLASTX alignments with the mouse genomic sequence. The GenomeScan and TWINSCAN results were obtained using their respective web servers. To obtain the GenomeScan predictions we uploaded for each input query sequence the complete protein sequences of all proteins that AGRIPPA used to generate *Protein or Combined *hints for AUGUSTUS, on average 23 proteins per sequence.

Table 1 summarizes the test results on sag178. AUGUSTUS predicts 42% of the genes correctly. The *EST *hints help to improve the accuracy. *Protein *hints improve the accuracy even more and the best accuracy was achieved by AUGUSTUS+ using *all *hints (option (*d*)). On these multi-gene sequences AUGUSTUS outperforms the other programs, especially on the gene level.

**Table 1:** Accuracy results on sag178

	**sag178**	base		exon		gene	
		sn	sp	sn	sp	sn	sp
	AUGUSTUS	0.93	0.83	0.79	0.73	0.42	0.38
	GENSCAN	0.94	0.64	0.68	0.45	0.18	0.14
	GENEID	0.89	0.78	0.67	0.60	0.17	0.17
	HMMGene	0.87	0.49	0.71	0.30	0.20	0.07

AUGUSTUS+	*EST*	0.95	0.85	0.85	0.76	0.49	0.46
	*Protein*	0.98	0.90	0.91	0.87	0.71	0.68
	*Combined*	0.95	0.89	0.89	0.85	0.68	0.65
	all hints	0.98	0.93	0.94	0.90	0.82	0.79
						
	GenomeScan*	0.83	0.77	0.69	0.64	0.37	0.38
	TWINSCAN	0.88	0.82	0.71	0.68	0.20	0.25

Accuracy results on human data set sag178 with 43 sequences and 178 genes, sn = sensitivity = TP/AP, and sp = specificity = TP/PP where TP, AP and PP are the number of true positives, actual positives and predicted positives, 'exon' and 'gene' here refer to the *protein-coding *parts of exons or genes, respectively. * For the accuracy values of GenomeScan the 7 sequences which are longer than 230 Kb were deleted from the test set. They were too long for the GenomeScan web server.

A more realistic test set is human chromosome 22. However, it is less completely and probably less accurately annotated than the above test set. As reference annotation of this test set, we used the 387 protein coding regions of all complete genes which have been published by Collins et al. [23]. These data were produced by the Chromosome 22 Group at the Sanger Institute and are available online along with the GENSCAN predictions [24].

Table 2 shows that, on Chromosome 22, AUGUSTUS is somewhat less sensitive than GENSCAN on the base and exon level but considerably more specific. Again, AUGUSTUS is more accurate than GENSCAN on the gene level. AUGUSTUS+ with all hints is in all measures the most accurate version of AUGUSTUS. It predicts 41% of the genes exactly as annotated. TWINSCAN and SGP2 are less accurate on the exon and gene level than the least accurate version of AUGUSTUS+ which uses *EST *hints alone. Again, the difference is especially demonstrative on the gene level. The accuracy of GenomeScan on chromosome 22 has been compared to that of GENSCAN and SGP2 by Parra et. al [5]. These authors conclude that "Overall, SGP2 appears to be more accurate than GENSCAN in human chromosome 22" and "GENOMESCAN [...] did not appear to be superior to GENSCAN in human chromosome 22.". Figure 1 shows how much the predictions of SGP2 and AUGUSTUS+ using *Combined *hints have in common with each other and with the annotation. The data of Figure 1 show, for example, that the intersection of the set of SGP2 exons and AUGUSTUS+ *Combined *exons would have 2271 true positive and only 439 false positives. Such a combined exon finder which predicts only the common exons of AUGUSTUS+ *Combined *and SGP2 would thus have an exon sensitivity of 0.65 and an exon specificity of 0.84.

To measure the advantage of interval hints over position wise hints we conducted the following two experiments where we compared AUGUSTUS+ to variants of it. First, we modified the set of *Protein *hints for the test set sag178 and replaced every hint of type *exon *or *exonpart *which goes from position *a *to *b *by an exonpart hint of length 1 at position (*a *+ *b*)/2 (rounded). We also removed the splice site hints but kept the *start *and *stop *hints. This is similar to the method of GenomeScan which uses a BLASTX hit to reward gene structures which include the so-called centroid of the hit in an exon. We reestimated the probabilities of these hints and obtained the following accuracy values on sag178: base sn = 93%, sp = 92%, exon sn = 85%, sp = 85%, gene sn = 62%, sp = 59%. We conclude that reducing each interval hint to a single point decreases the overall accuracy. Further we tried the following variant. We removed the hints of type *start, stop, ass *and *dss *and replaced very hint of type *exon *or *exonpart *which goes from position *a *to *b *by a set of *b *- *a *+ 1 *exonpart *hints each of length 1, the positions ranging from *a *to *b*. This variant yields: base sn = 98.5%, sp = 86%, exon sn = 84%, sp = 79%, gene sn = 53%, sp = 49%. This indicates that the common model solution of upvaluing gene structures independently for every coding base that overlaps the database match is not optimal.

**Table 2:** Accuracy results on chromosome 22

	**chr22**	base		exon		gene	
		sn	sp	sn	sp	sn	sp
	AUGUSTUS	0.85	0.59	0.71	0.52	0.19	0.09
	GENSCAN	0.89	0.49	0.73	0.39	0.10	0.05

AUGUSTUS+	*EST*	0.90	0.63	0.80	0.58	0.25	0.12
	*Protein*	0.94	0.65	0.87	0.63	0.37	0.19
	*Combined*	0.91	0.65	0.81	0.61	0.30	0.15
	all hints	0.94	0.68	0.89	0.66	0.41	0.22
						
	TWINSCAN	0.85	0.65	0.76	0.58	0.14	0.10
	SGP2	0.87	0.66	0.74	0.56	0.19	0.10

Accuracy results on human chromosome 22 using the annotation of the Sanger Center as a standard of truth. The results were computed using the program Eval from Evan Keibler and Michael R. Brent. The TWINSCAN and the SGP2 predictions were taken from [28] and [29]. The AUGUSTUS predictions can be downloaded from the AUGUSTUS web server [27]

To estimate the magnitude of the malus effect and to obtain an upper bound on the number of genes that may be missed because there is no extrinsic evidence for them we performed the following experiment. On chromosome 22 we used the AUGUSTUS+ version that uses *EST *hints but gave AUGUSTUS+ an empty set of hints. So the model 'thought' that an EST database search has been performed on that sequence but the search has produced no results. Compared to the *ab initio *predictions the number of predicted exons decreased from 4824 to 4183. However, the percentage of annotated genes whose coding sequence overlaps a predicted coding sequence decreased only very little, from 93% to 92%. The accuracy results are: base sn = 83%, sp = 63%, exon sn = 68%, sp = 57%, gene sn = 19%, sp = 10%. On the base and exon level the sensitivity decreased a little but the specifity even increased more than the sensitivity decreased. We conclude that only exons that are very doubtfull according to the intrinsic evidence are not predicted when extrinsic evidence is missing. The effect is still weak enough so that the gene level sensitivity remains the same.

## Discussion

In this paper, we introduced a novel *Generalized Hidden Markov Model *for gene prediction that integrates intrinsic and extrinsic information in one single probabilistic model. When considering hints from extrinsic sources in addition to intrinsic sequence features, it is crucial to incorporate these hints in a probabilistic manner that allows to ignore hints which disagree with strong intrinsic evidence. A major difficulty in combining extrinsic and intrinsic information is to find a reasonable balance between these different sources of information. We solved this problem by introducing a new probabilistic approach that incorporates both intrinsic and extrinsic evidence in one stochastic model.

In our program evaluation, we compare the *quality *of the respective software programs in terms of their prediction accuracy. When comparing the accuracy of extrinsic methods, one should keep in mind that AUGUSTUS+ and GenomeScan use different sources of external information (EST and protein or protein only) than the programs TWINSCAN and SGP2 do (genomic sequences). With this evaluation it is not possible to directly compare the efficiency of the underlying methods for integrating extrinsic information. Our approach allows for *exonpart *hints that indicate that a certain region or position is coding as well as for *exon *hints that indicate the location of a *complete *exon. An *exon *hint gives a bonus to an exon candidate only if both the begin and end position are confirmed by an alignment to EST or protein sequences. This is in contrast to the integration methods of GenomeScan and HMMGene, where the gene structure gets bonuses, if it classifies certain positions as coding. We believe that, in the case when a putative *complete *exon can be inferred by an EST alignment to the genomic sequence, a considerable amount of information is lost by reducing such a hint to a set of hints to *individual *positions. We think that this is one reason why the use of ESTs alone increases the accuracy of AUGUSTUS+ in multi-gene sequences but not the accuracy of HMMGene.

An implication of the here described extension of a GHMM is that an *unsuccessful *search for hints in a certain region makes exons in this region *less likely*. This is not the case for HMMGene and GenomeScan. If the *ab initio *versions of these programs predicts an additional false exon in an otherwise correctly predicted gene then the extended versions of the programs that use extrinsic information, will also predict the false positive exon. This is so even if a very sensitive method of finding extrinsic information has produced no results supporting that false positive exon. By contrast, our method would give a *malus *to a possible exon that is not supported by the extrinsic information. Thus, with our model the non-supported false positive exon is less likely to be included in the output gene model if extrinsic information is used. Whether the malus defect is desirable depends on the objective when predicting genes. If the aim is to make the best guess on the gene structure of a sequence, based on both sequence intrinsic and extrinsic evidence then in the spirit of Bayesian statistics a lack of extrinsic evidence lowers the likelyhood of genes and the malus effect is desirable. If however the aim is to explicitly find 'new' genes without homology evidence, then an *ab initio *gene finder is the method of choice. The objective will be different for different research applications of the same genome project and a solution is to have different sets of gene predictions. The method that we described can be readily applied to hints coming from other sources than EST and protein databases. We currently develop an approach to generate hints coming from cross-species alignments of genomic sequences. To this end, we use a previously described combination of the alignment programs DIALIGN [25] and CHAOS [26] to generate *exonpart *hints by locating segments of the input genomic sequence with high similarity on the peptide level to segments of a genomic sequence from a related species. As another possible extension of the AUGUSTUS model, one could include states for exons and introns in the non-coding region. This way, the current systematic error of assuming that *all *EST matches are likely to be in coding regions can be corrected. Many more approaches are possible to integrate extrinsic information in our GHMM. We will explore these approaches systematically in the future to further improve the predictive power of AUGUSTUS+.

## Availability and requirements

AUGUSTUS+ is an option to the program AUGUSTUS. The source codes of AUGUSTUS and AGRIPPA are freely available for download from the AUGUSTUS web server [27]. Both programs are written in C++ and have been successfully compiled under Linux, UNIX and Mac. The augustus web server also hosts a web interface to AUGUSTUS including the possibility of giving user-defined constraints.

## Authors' contributions

Mario Stanke developed the model together with Stephan Waack, implemented AUGUSTUS and the model extension and conducted the performance testing. Oliver Schöffmann implemented the program AGRIPPA. Burkhard Morgenstern prepared the manuscript together with Mario Stanke. Stephan Waack developed the model together with Mario Stanke.
